# Optimizing Procedures of Ultrasound-Assisted Extraction of Waste Orange Peels by Response Surface Methodology

**DOI:** 10.3390/molecules27072268

**Published:** 2022-03-31

**Authors:** Chao-Hui Feng

**Affiliations:** 1 School of Regional Innovation and Social Design Engineering, Faculty of Engineering, Kitami Institute of Technology, 165 Koen-cho, Kitami 090-8507, Hokkaido, Japan; feng.chaohui@mail.kitami-it.ac.jp; 2 RIKEN Centre for Advanced Photonics, RIKEN, 519-1399 Aramaki-Aoba, Aoba-ku, Sendai 980-0845, Miyagi, Japan

**Keywords:** hesperidin, response surface methodology, ultrasound-assisted extraction, waste orange peels

## Abstract

The simultaneous effects of three continuous factors: solvent concentration (50–100%), treated times (25–85 min), treated temperatures (25–55 °C), and two categorical factors: type of solvents (methanol or ethanol) and ultrasonic frequency (28 kHz or 40 kHz) on ultrasonic-assisted extraction yield from waste orange peels were evaluated and optimized by response surface methodology. Fourier Transform Infrared (FTIR) spectroscopy with a wavelength of 500 cm^−1^ to 4000 cm^−1^ was employed to rapidly identify the orange extracts. The significant polynomial regression models on crude extraction, sediments after evaporation, and precipitation yield were established (*p* < 0.05). Results revealed that solvent concentration affected crude extraction and precipitation yield linearly (*p* < 0.01). The optimal and practical ultrasound-assisted extraction conditions for increasing the precipitation yield were using 61.42% methanol with 85 min at 55 °C under 40 kHz ultrasonic frequency. The spectra of extracts showed a similar fingerprint of hesperidin.

## 1. Introduction

The peels from citrus fruit possess a large number of bioactive compounds, such as flavonoids and phenolic acids. As one of the most important groups of dietary phenolics, a variety of flavonoids cannot be synthesized biologically [1]. However, as the secondary metabolism compounds, they widely exist in the plant kingdom such as vegetables, fruits, grains, green leaves, and so on [2]. Besides antioxidant, antiviral, and antimicrobic properties, flavonoids have been discovered to potentially inhibit coronaviruses [3]. It was reported that flavonoids such as hesperidin and rutin had a better binding affinity to the main protease of COVID-19 than nelfinavir [3]. Wu et al. (2020) stated that hesperidin possessed the most suitable substance to bind to the “spike” of SARS-CoV-2 after testing 1066 natural substances with potential antiviral effects and 78 antiviral drugs [4]. Other authors stated that it was easy to bind hesperidin with SARS-CoV-2 due to lower energy being required [3,5]. In this way, hesperidin prevents the virus to bind with the host cell. Approximately 400 thousand tons of fruits juice is consumed in Japan per year and approximately 1000 tons of orange waste is produced in Shizuoka prefecture in Japan [6]. Currently, the prices for hesperidin, naringin, and neohesperidin are sold approximately as USD 192, USD 487, and USD 142,599 per 100 g in Japan, respectively. It is, thus, useful to extract flavonoids from waste orange peels, which can not only maximize the reuse and reduce environmental problems [7], but also exploit the value-added by-products and cut down industrial economical costs [8].

Like supercritical extraction [9], microwave-assisted extraction [10], and pressurized liquid extraction [11], ultrasound-assisted extraction (UAE) is also regarded as the eco-friendly green extraction technology [12]. It has been widely utilized to extract bioactive compounds from citrus waste due to its much shorter extraction time, lower energy consumption, and safe thermolabile constituents [13,14]. 

Response surface methodology (RSM), which estimates the effects of many factors and their interactions on response variables, has been comprehensively utilized to model and optimize food processes [15,16,17,18,19]. The extraction conditions of phenolic compounds from orange peels using RSM were investigated and the optimal ultrasound extraction time, temperature, and ethanol concentration were reported to be 44 min, 50 °C, and 57.7%, respectively [13]. The total phenolic content was reported to be 292.158 µg catechol/g and the total flavonoid content was 191.144 µg catechol/g under those optimal conditions [13]. The extraction of polysaccharides from *Momordica charabtia* L. was developed by enzymolysis-ultrasonic-assisted extraction and optimized using Box-Behnken design [19]. Pectin from sour orange peel was done by using UAE and the maximum extraction yield (28.07 ± 0.67%) can be achieved when the ultrasound power of 150 W with 10 min was employed under the pH of 1.5. RSM can interpret the relationship between the responses [20] and variables and greatly decreases the number of experiments in comparison with the full factorial experimental design [13,18].

Fourier Transform Infrared (FTIR) spectroscopy, like hyperspectral imaging [21,22,23,24], Raman spectroscopy [25], terahertz spectroscopy [6,26], and NIR images [27], plays an important role in detecting foodstuffs [28]. As variations in the permanent dipoles will lead to a specific vibrational mode occurring, two vibrations related to molecular bonds will stretch and bend [28]. The absorption in the IR range will occur at the feather frequencies, which can be used for identifying the presence of numerous chemical groups [28]. The interactions of hesperidin and naringin with dimyristoylphosphatidylcholine (DMPC) were studied using attenuated total reflection Fourier transform infrared (ATR-FTIR) spectroscopy, differential scanning calorimetry (DSC), atomic force microscopy (AFM), and field emission scanning electron microscopy (FE-SEM) [29]. Results reveal that hesperidin showed a weakly disordering effect in the hydrophobic region, while naringin possesses an ordering effect in this region [29]. 

All those studies indicate an unyielding interest in extracting bioactivities more efficiently. Although previous studies have studied the effects of different ultrasound-assisted extraction conditions (different ethanol concentrations, temperatures) on total phenolic content, total flavonoid content, and antioxidant activities by RSM [13], the simultaneous effects of two different types of solvents and ultrasonic frequency as two categorical factors, three continuous factors like solvent concentration, treated times, and treated temperatures on precipitation yield have not been exploited. This information could be used for improving the precipitation yield and maximizing the profit for the reuse of the waste orange peels.

The objective of this study is thus to evaluate the effects of different solvents, ultrasonic powers, temperatures, times, and solvent concentrations on precipitation yield from the waste orange peels. Following this, extracts will be detected by FTIR. The most optimal extraction processing will be established by using RSM. 

## 2. Results and Discussion

### 2.1. Crude Extraction

Nowadays, the safe extraction of natural bioactive from industrial waste has drawn great attention, as those byproducts can be applied to functional foods and nutraceutical applications [8,30,31]. The regression model developed for crude extraction was statistically significant with an R^2^ value of 80.49% (*p* < 0.05). The lack of fit for this regression model was not significant (*p* > 0.05), and thus, it was highly adequate (Table 1). An R^2^ value between 66% and 81% is regarded as an acceptable level, while an R^2^ value over 91% is believed to be an excellent prediction for quantitative prediction [32,33]. According to the current experimental design, the predicted polynomial regression equations for crude extraction (Y_1_) as a function of solvent concentration (X_1_), treated times (X_2_), and treated temperatures (X_3_) for ethanol with the ultrasonic frequency of 28 kHz in the uncoded units are as follows:

Y_1_ = 63.6 − 0.789X_1_ + 0.394 X_2_ + 0.304X_3_ + 0.00074 X_1_ X_2_ + 0.00568 X_1_ X_3_ − 0.00778 X_2_ X_3_ (1)

The equations for methanol with an ultrasonic frequency of 28 kHz in the uncoded units are as follows:

Y_1_ = 82.7 − 0.925X_1_ + 0.469 X_2_ + 0.089X_3_ + 0.00074 X_1_ X_2_ + 0.00568 X_1_ X_3_ − 0.00778 X_2_ X_3_ (2)

The equations for ethanol with an ultrasonic frequency of 40 kHz in the uncoded units are as follows:

Y_1_ = 109.6 − 0.972X_1_ + 0.085 X_2_ + 0.061X_3_ + 0.00074 X_1_ X_2_ + 0.00568 X_1_ X_3_ − 0.00778 X_2_ X_3_ (3)

The equations for methanol with an ultrasonic frequency of 40 kHz in the uncoded units are as follows:

Y_1_ = 127.0 − 1.108X_1_ + 0.159 X_2_ − 0.154X_3_ + 0.00074 X_1_ X_2_ + 0.00568 X_1_ X_3_ − 0.00778 X_2_ X_3_ (4)

Solvent concentration affected the crude extraction linearly (*p* < 0.01) (Table 1). Based on the corresponding coefficient, it can be observed that the effects of solvent concentration were augmented with an increase of the ultrasonic frequency. The effects of solvent concentration on methanol were greater than that on ethanol. It was reported that the highest extraction yield from the sweet orange peel was obtained using methanol extract (15.56 ± 0.60 g/100 g) [31]. Methanol was concluded as more efficient in extracting phytochemicals from citrus peels than other organic solvents, such as hexane, petroleum ether, and acetone [31]. The polarity of the extraction solvents may attribute to this phenomenon. A higher extraction yield was occurred in methanolic extract, implying that a highly polar solvent facilitates the extraction efficiency [34]. The relative polarities of methanol and ethanol are 0.762 and 0.654, respectively. Truong et al. (2019) also observed a higher extraction yield achieved in methanolic extraction and attributes this observation to the higher solubility of phenolics, flavonoids, alkaloids, and terpenoids in methanol than the other solvents, such as ethanol [34]. The negative sign implies that an increase in solvent concentration will decrease the crude extraction. The hesperidin extraction yield from honeybush (Cyclopia maculata) tea by using different concentrations of ethanol from 0% to 100% (*v*/*v*) was studied by Du preez et al. (2016) [35]. The highest hesperidin content was achieved by using 58% ethanol [35]. Feng et al. (2020) also found that 0.060 ± 0.070 mg/40 g dried orange hesperidin and 0.015 ± 0.018 mg/40 g dried orange naringin could be obtained using 80% ethanol, while no flavonoids could be obtained using 100% ethanol [8]. Barrales et al. (2018) found that there was no considerable difference between the samples extracted by 75% and by 50% ethanol concerning the yield of hesperidin extraction [36]. Regarding the current study, the highest crude extraction (77.6 g per 10 g) was obtained when 50% ethanol along with 40 kHz of ultrasonic frequency at 25 °C were applied for 55 min (Table 2). The increased extraction yield in a lower concentration may be due to the enhancement of solubility of chemicals in the combination of water and organic solvent. Do et al. (2014) studied the effects of different concentrations (50%, 75%, and 100%) of methanol, ethanol, and acetone on extracting bioactive compounds of *Limnophila aromatica* and observed that the extraction yield increased with the increasing of water concentration in the solvent (i.e., lower concentration) [37]. It was concluded that the combined utilization of organic solvent and water was able to enhance the exactions of chemicals that are soluble in water and/or organic solvent [37]. The higher crude extraction weight obtained from the lower solvent concentration than the weight of initial orange peels is probably due to the weight of the water left in the extracts. 

**Figure 1:**
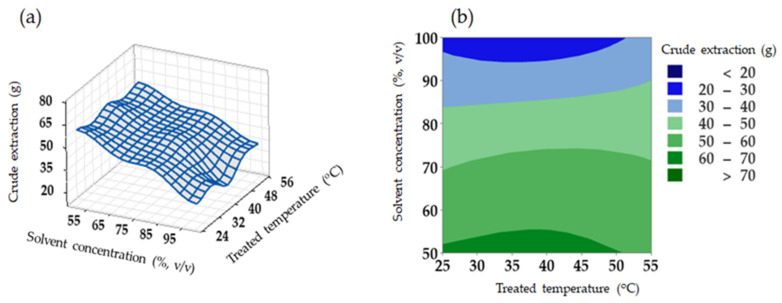
Response surface plot (**a**) and 2D contour plot (**b**) for the effect of solvent concentration and treated temperature on crude extraction.

The interactive effect of solvent concentration and treated temperature (*p* < 0.05) was observed in Figure 1. Higher crude extraction can be achieved when a lower concentration with a higher temperature were applied (Figure 1a). According to the 2D contour plot (Figure 1b), the crude extraction can reach over 60 g if the solvent concentration was lower than 70%. A higher ethanol concentration was able to facilitate hesperidin extraction because of the higher solubility in the solvent. However, it may not occur when extracting a very polar solute [35]. If water mixes with ethanol or methanol, water can bind the cell wall, leading to swollen plant matrix. Nevertheless, ethanol can disrupt the bonding of solutes of plant matrices [38]. The addition of water to ethanol could increase the recovery of glycosylated flavonoids [36]. The ultrasound-assisted extraction of flavonoids from grapefruit (*Citrus paradisi L.*) solid wastes was optimized by RSM [39]. Results show that an increase in temperature improved the extraction of flavonoids, but a high ethanol concentration did not improve the extraction. It was explained that the presence of ethanol in the solvent has a positive effect on the polyphenol extraction when a maximum ethanol concentration is reached, from which the polyphenol extraction decreases. Ethanol affects the reduction of the dielectric constant of the solvent, leading to the increased solubility and diffusion of polyphenols [39]. However, a highly pure organic solvent may result in the dehydration and collapse of the vegetable cells and cause the denaturation of cell wall proteins, restraining the polyphenols diffusion to the extracting liquid [39]. Temperatures can improve mass transfer during extraction [39,40]. As a result, a lower concentration associated with a higher temperature in the current study can achieve a higher crude extraction. 

### 2.2. Sediments after Evaporation and Left Overnight

The regression model developed for sediments after evaporation and left overnight (Y_2_) was significant (*p* < 0.05) with an R^2^ value of 77.01%. The polynomial regression models for ethanol with ultrasonic frequency of 28 kHz in the uncoded units are as follows:

Y_2_ = 30.8 − 0.490 X_1_ + 0.508 X_2_ + 0.859 X_3_ − 0.00034 X_1_X_2_ − 0.00440 X_1_X_3_ − 0.00842 X_2_X_3_ (5)

The polynomial regression models for methanol with ultrasonic frequency of 28 kHz in the uncoded units are as follows:

Y_2_ = 22.3 − 0.434 X_1_ + 0.489 X_2_ + 0.962 X_3_ − 0.00034 X_1_X_2_ − 0.00440 X_1_X_3_ − 0.00842 X_2_X_3_ (6)

The polynomial regression models for ethanol with ultrasonic frequency of 40 kHz in the uncoded units are as follows:

Y_2_ = 64.1 − 0.521 X_1_ + 0.232 X_2_ + 0.637 X_3_ − 0.00034 X_1_X_2_ − 0.00440 X_1_X_3_ − 0.00842 X_2_X_3_ (7)

The polynomial regression models for methanol with ultrasonic frequency of 40 kHz in the uncoded units are as follows:

Y_2_ = 51.2 − 0.464 X_1_ + 0.214 X_2_ + 0.740 X_3_ − 0.00034 X_1_X_2_ − 0.00440 X_1_X_3_ − 0.00842 X_2_X_3_ (8)

Again, the sediments after evaporation and left overnight were influenced by solvent concentration linearly (*p* < 0.01) (Table 1). A higher ultrasonic frequency (40 kHz) increases the effects of ethanol concentration (higher absolute coefficient value: 0.521) on sediment yield. Ultrasound waves, ranging from 20 kHz to 100 MHz, can damage and disrupt cell membranes during extraction. In this way, the cell contents will be released, and thus, increase the extraction yield [13]. On the other hand, the boiling point of ethanol (78.4 °C) is higher than that of methanol (64.7 °C); after the same vacuum evaporation for eliminating the organic solvents, there was more ethanol left in the extraction solution, leading to the comparably higher weight and sediments yield.

### 2.3. Precipitation Yield

According to a previous study, precipitation contains certain useful bioactive compounds, such as hesperidin, naringin, quinic acid, p-coumaric acid, and so on [8]. Consequently, the precipitation yield is essential from the industrial economical point of view. It is environmentally friendly and of practical use for optimizing the extraction of those bioactive compounds from waste orange peels. The regression model developed for Y_3_ was significant (*p* < 0.05), although the R^2^ was not high (R^2^ = 53.06%).

**Figure 2:**
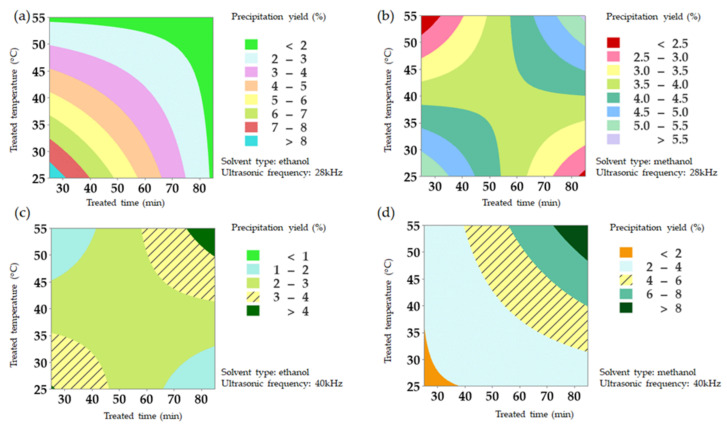
Contour plot for the effect of treated time and treated temperature on precipitation yield under different solvent types [(**a**,**c**): ethanol; (**b**,**d**): methanol] and ultrasonic frequencies [(**a**,**b**): 28 kHz; (**c**,**d**): 40 kHz].

Figure 2 illustrates the interactive effects of treated time and temperature (*p* < 0.01). The two-way interaction showed significance at a 1% level and its contribution occupied nearly half (36.72%) of that for the whole model (52.95%) (Table 1). Take the threshold of 75% solvent concentration as an example, higher precipitation yield (>4%) achieved with a shorter treated time (<60 min) with a lower temperature (<45 °C) for samples extracted by ethanol at 28 kHz (Figure 2a). 

A longer treated time (>60 min) combined with a higher treated temperature (>45 °C) or a shorter treated time (<50 min) with a lower treated temperature (<35 °C) rendered a higher precipitation yield (>4%) concerning methanol at 28 kHz (Figure 2b). 

Concerning a higher ultrasonic frequency (40 kHz), a higher temperature (>45 °C) along with a longer treated time (>60 min) leads to a higher precipitation yield (>3%) for samples extracted by ethanol (Figure 2c) and methanol (Figure 2d). 

### 2.4. Processing Optimization

To achieve a higher precipitation yield, two predictive modules were set up. Module 1: the goal of the precipitation yield was set as maximum (regardless of other response parameters) and the predicted precipitation yield would be 11.95%. The results of the optimization were using 100% methanol with 85 min at 55 °C under 40 kHz ultrasonic frequency. Module 2: Y_3_ was set as the most important parameter, followed by Y_1_ and Y_2_ (less importance), the precipitation yield was predicted to be 8.30%. Here, the optimized operational parameters of solvent concentration, treated times, treated temperature, type of solvents, and ultrasonic frequency were 61.42%, 85 min, 55 °C, methanol, and 40 kHz, respectively. To validate the optimized operational conditions provided by the modules, the two additional laboratory works were conducted in triplicates. The experimental precipitation yields for Modules 1 and 2 were 17.19 ± 1.03% and 6.29 ± 1.26%, respectively. Module 2 is recommended for economically and more practical industrial utilization of solvent concentration.

### 2.5. Spectral Characteristics Overview for the Sample with Different Treatments Measured by FTIR

**Figure 3:**
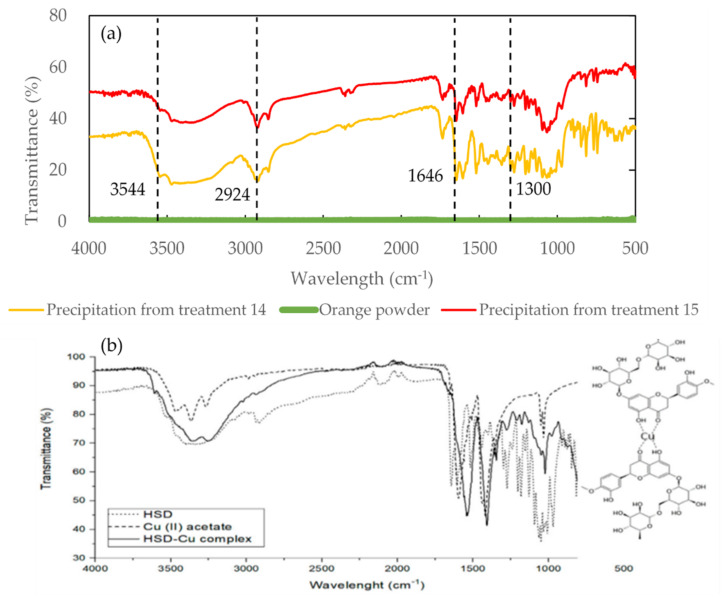
The transmittance of precipitation with different ultrasonic treatments mixed with potassium bromide detected by FTIR (**a**) and hesperidin–copper complex cited from the study of Stanisic et al. (2020) [41] (**b**).

Figure 3 illustrates transmittance spectra of an orange powder: precipitations from different treatments detected by using FTIR. There was no absorption peak for the sample before treatment (i.e., orange powder). No considerable differences between the sample with treatment 14 (sample extracted by 100% methanol with 55 min at 25 °C using 40 kHz) and 15 (sample extracted by 75% ethanol with 85 min at 25 °C using 28 kHz) were observed in accordance with their spectra in the infrared range. Further investigation displays that extract from orange peels (Figure 3a) possessed a similar fingerprint of hesperidin (Figure 3b) in the wavelength from 500 cm^−1^ to 4000 cm^−^^1^ [41], indicating that it contains hesperidin. 

It was stated that the vibration stretching for -C=O in hesperidin was at 1644 cm^−1^ and the absorption peaks for C-O were detected at 1297, 1275, 1242, 1206, 1184, 1154, 1132, 1095, 1054, 1037, and 1009 cm^−1^ [41]. The absorption peaks detected at 3544 and 2924 cm^−1^ (Figure 3) may be related to hydroxyl (-OH) [42] and aliphatic (CH) [43] stretching vibration for hesperidin. The IR spectra of hesperidin were also reported to present the characteristic patterns of flavonoids at 1649 cm^−1^ (C=O-valence), 3545 cm^−1^ (-OH-valence), 2938 cm^−1^ (CH-valence, arene), 2917 cm^−1^ (-CH valence, alkane), and 2850–2865 cm^−1^ (-CH valence), and characteristics patterns of methoxylic (alkane, OCH_3_) at 1277 cm^−1^ [44].

The spectra of hesperidin and naringin in the range of 2800–3000 cm^−1^ were studied via employing ATR-FTIR [29] and it is difficult to identify the differences in this range due to the similar molecule structure [8]. However, the fingerprint spectrum of hesperidin was able to clearly distinguished from that of naringin in the terahertz range [6]; thus, it is interesting to detect samples with different ultrasonic treatments by THz spectroscopy to verify whether there is any difference.

The treatment 14 sample showed a lower transmittance (i.e., higher absorbance) than the treatment 15 sample, indicating a higher concentration of hesperidin content in the precipitation due to the saturation effect. 

## 3. Materials and Methods

### 3.1. Sample Preparation and Ultrasound-Assisted Extraction

The peels of sweet oranges (*Citrus sinensis*) were dried in an oven (40 °C) for 7 days. The dried orange peels were blade milled and an average of 10.05 ± 0.08 g orange powder, mixed with 200 mL of solvent, was put in a flask to an ultrasonic bath with the internal dimensions of 30.0 cm × 24.0 cm × 15.0 cm and a capacity of 10 L (MCD-10P, ASONE Corporation, Osaka, Japan). The sample was centrifuged at 4000 rpm for 15 min at 20 °C. The supernatant was collected and a rotary vacuum evaporator (EYELA NVC-2100, Rikakikai Co. Ltd., Tokyo, Japan) was used to eliminate the excess solvent at 45 °C at 61 hPa for 8 min (Y_1_). The extracts were left overnight to continuously render the solvent evaporate throughout (Y_2_).

The extracts from the UAE were diluted with distilled water at twice the weight of the extract. The sediments were vacuum filtered with filter paper and stored in the desiccator. The precipitation yield (Y_3_) was calculated as:

(9) $$Y_{3}\;=\;\frac{W_{p}}{W_{o}}\;\times \;100\%$$

where W_p_ and W_o_ were the weight of precipitate and orange powder, respectively. The extraction method was modified based on the methods of Wang et al. [45], Shehata et al. [13], and Feng et al. [8].

### 3.2. Experiment Design

The simultaneous effects of three continuous factors and two categorical factors on the yield of extraction were studied using RSM. The three continuous factors were solvent concentration (X_1_: 50–100%), treated times (X_2_: 25–85 min), and treated temperatures (X_3_: 25–55 °C), while the two categorical factors were types of solvents (X_4_: methanol (Lot. DLM3136, FUJIFILM Wako Pure Chemical Corporation, Osaka, Japan) or ethanol (Lot. DLM2697, FUJIFILM Wako Pure Chemical Corporation, Osaka, Japan)) and ultrasonic frequency (X_5_: 28 kHz or 40kHz). A Box-Behnken design (BBD) [45] was employed and performed in Minitab 21.1 software (Kozo Keikaku Engineering Inc., Tokyo, Japan). The uncoded values are displayed in Table 2 and a total of 60 experiments consisted of the whole design. The responses (Y_n_; n = 1–3) in this study were crude extraction after UAE (Y_1_), sediments after evaporation and left overnight (Y_2_), and the yield of the precipitation (Y_3_). The experimental data were fitted to a second-order polynomial model below:

(10) $$\mathrm{Yn}\;=\alpha_{0}\;+\;\sum_{i=1}^{3}\alpha_{i}x_{i}\;+\;\sum_{i=1}^{2}\sum_{j=i+1}^{3}\alpha_{\mathrm{ij}}x_{i}x_{j}$$

where $\alpha_{0}$ was the constant of the model and $\alpha_{i}$ and $\alpha_{ij}$ were the linear and interaction coefficients, respectively. To minimize the effect of unexplained variability in the observed responses due to extraneous factors, all the experiments were carried out in a randomized order.

The three-dimensional contour curve of the response surface and all the coefficients of the polynomial model were calculated using Minitab 21.1. The significance of the regression parameters for a response was estimated by the F-test (*p <* 0.05). The determination coefficient (R^2^) and non-significant lack of fit were utilized to estimate the accuracy of fitted models. 

### 3.3. Compounds Pellets Preparation and FTIR Analysis

Anhydrous potassium bromide (KBr, Jasco Corporation, Tokyo, Japan), as a background, was well ground and the orange powders and flavonoids extracts were mixed with KBr. The mixture was put into a transparent disc (as a pellet holder) and placed onto an evacuable die. The pellets were made by pressing the die for 10 s. The spectra were detected and recorded by using an FTIR spectroscopy (FT/IR-4700, Jasco, Tokyo, Japan) with a rapid scanning speed of 16 cm^−1^ and a resolution of 0.4 cm^−1^.

## 4. Conclusions

The current study successfully applied RSM to elaborate the simultaneous effects of three continuous factors and two categorical factors on ultrasound-assisted extraction yield. The optimal extraction conditions were established and could provide useful information for food and pharmaceutical applications. A deeper investigation shows that solvent concentration played an important role in crude extraction. Interactive effects of treated time and treated temperature influenced the precipitation yield and precipitation contained hesperidin. RSM can effectively optimize extraction conditions and the ultrasonic extracts are recommended to be identified in the terahertz range in future work.

## Figures and Tables

**Table 1:** Regression coefficients and analysis of variance of the regression models for extraction efficiency.

Source of Variation	Df	Response Variables								
		Crude Extraction (Y_1_, g)			Sediments after Evaporation and Left Overnight (Y_2_, g)			Precipitation Yield (Y_3_, %)		
Source		SSS	Contribution (%)	F	SSS	Contribution (%)	F	SSS	Contribution (%)	F
Model	15	11,284.10	80.49	12.10 **	10,569.60	77.01	9.83 **	2.64	52.95	3.30 **
Linear	5	9820.60	70.05	31.60 **	9546.90	69.56	26.63 **	0.81	16.34	3.07 *
X_1_ (%, *v*/*v*)	1	9248.00	65.97	148.78 **	9035.00	65.83	126.02 **	0.67	13.56	12.71 **
X_2_ (min)	1	12.90	0.09	0.21 ^ns^	0.10	0.00	0.00 ^ns^	0.00	0.08	0.09 ^ns^
X_3_ (°C)	1	38.90	0.28	0.63 ^ns^	0.30	0.00	0 ^ns^	0.00	0.01	0.01 ^ns^
X_4_ (methanol or ethanol)	1	189.40	1.35	3.05 ^ns^	166.30	1.21	2.32 ^ns^	0.12	2.33	2.20 ^ns^
X_5_ (kHz)	1	331.40	2.36	5.33 *	345.10	2.51	4.81 *	0.02	0.36	0.33 ^ns^
2-Way Interaction	10	1463.50	10.40	2.35^*^	1022.70	7.45	1.43 ^ns^	1.82	36.72	3.42 **
X_1_×X_2_	1	5.00	0.04	0.08 ^ns^	1.10	0.01	0.01 ^ns^	0.03	0.57	0.54 ^ns^
X_1_×X_3_	1	72.70	0.52	1.17 *	43.60	0.32	0.61 ^ns^	0.01	0.12	0.12 ^ns^
X_1_×X_4_	1	92.50	0.66	1.49 *	15.80	0.12	0.22 ^ns^	0.18	3.53	3.33 ^ns^
X_1_×X_5_	1	167.40	1.19	2.69 ^ns^	4.60	0.03	0.06 ^ns^	0.00	0.01	0.01 ^ns^
X_2_×X_3_	1	196.00	1.40	3.15 ^ns^	229.50	1.67	3.20 ^ns^	0.46	9.25	8.61 **
X_2_×X_4_	1	40.30	0.29	0.65 ^ns^	2.40	0.02	0.03 ^ns^	0.28	5.57	5.22 *
X_2_×X_5_	1	689.10	4.92	11.09 **	547.00	3.99	7.63 *	0.30	6.02	5.55 *
X_3_×X_4_	1	82.90	0.59	1.33 ^ns^	18.90	0.14	0.26 ^ns^	0.24	4.91	4.56 *
X_3_×X_5_	1	106.20	0.76	1.71 ^ns^	88.40	0.64	1.23 ^ns^	0.30	6.02	5.58 *
X_4_×X_5_	1	11.40	0.08	0.18 ^ns^	71.50	0.52	1.00 ^ns^	0.04	0.72	0.65 ^ns^
Error	44	2734.90	19.50		3154.70	22.99		2.33	46.94	
Lack-of-Fit	36	2518.90	17.97	2.59 ^ns^	2750.40	20.04	1.51 ^ns^	2.32	46.62	31.75 **
Pure Error	8	216.10	1.54		404.30	2.95		0.02	0.32	
Total	59	14,019.00	100.00		13,724.30	100.00		4.97	100.00	
R^2^(%)			80.49			77.01			53.06	

Note: ** means significant at *p ≤* 0.01; * means significant at *p ≤* 0.05. ^ns^ means not significant. SSS: sequential sum of squares; F: ratio of variance estimates; Df: degree of freedom; X_1_: solvent concentration; X_2_: treated times; X_3_: treated temperatures; X_4_: types of solvents; X_5_: ultrasonic frequency.

**Table 2:** Matrix of Box-Behnken design for treatment combinations and the response of crude extraction, sediments after evaporation and left overnight, and the yield of the precipitation.

Treatment	X_1_ (%)	X_2_ (min)	X_3_ (°C)	X_4_	X_5_ (kHz)	Y_1_ (g)		Y_2_ (g)		Y_3_ (%)	
						Experimental	Predicted	Experimental	Predicted	Experimental	Predicted
1	100	25	40	Ethanol	40	24.30	33.80	1.50	16.45	3.71	2.78
2	75	25	25	Methanol	40	53.40	51.21	22.90	26.12	2.61	1.86
3	50	85	40	Methanol	28	66.30	67.92	40.00	41.84	1.66	1.35
4	50	25	40	Methanol	40	67.50	73.92	40.20	45.33	2.32	0.32
5	75	85	55	Ethanol	28	44.00	46.43	32.30	24.76	2.50	1.65
6	75	85	25	Methanol	40	65.00	52.44	31.30	24.79	2.30	2.50
7	75	55	40	Ethanol	40	46.90	46.81	30.00	30.17	2.61	2.69
8	50	55	25	Ethanol	40	77.60	65.64	55.00	48.74	2.03	1.70
9	50	85	40	Methanol	40	66.30	67.04	36.80	36.94	1.39	3.48
10	100	55	25	Ethanol	40	30.90	26.2	37.40	16.27	2.13	3.41
11	100	55	55	Methanol	40	22.60	28.31	4.40	5.74	5.32	7.85
12	50	55	25	Methanol	40	68.10	74.94	44.60	40.28	1.25	-0.16
13	75	25	25	Methanol	28	36.30	34.45	13.70	11.91	4.14	5.54
14	100	55	25	Methanol	40	34.50	28.7	13.40	10.63	3.36	4.52
15	75	85	25	Ethanol	28	51.00	44.36	43.30	30.35	1.96	1.84
16	75	85	55	Methanol	40	47.00	40.78	20.70	15.63	13.81	9.59
17	100	85	40	Methanol	40	15.50	26.18	3.70	3.47	9.18	8.60
18	75	55	40	Ethanol	40	37.80	46.81	14.80	30.17	3.33	2.69
19	50	55	25	Ethanol	28	48.80	51.85	36.30	37.68	1.52	4.34
20	50	25	40	Ethanol	40	67.80	70.09	50.40	51.71	1.41	2.30
21	50	85	40	Ethanol	40	52.30	58.72	39.60	44.4	1.93	1.75
22	50	55	55	Methanol	40	63.60	66.02	45.20	41.99	2.36	3.96
23	100	85	40	Ethanol	40	15.00	24.65	1.40	8.12	4.62	3.92
24	75	25	25	Ethanol	40	49.70	47.55	41.80	32.63	2.02	4.08
25	100	55	25	Ethanol	28	25.10	21.56	1.60	6.72	3.50	6.23
26	100	25	40	Methanol	28	23.70	22.3	5.80	2.77	7.82	5.62
27	75	25	55	Ethanol	28	41.90	45.12	28.80	23.62	3.52	1.80
28	75	55	40	Methanol	28	59.80	45.67	33.30	22.05	2.57	3.90
29	50	25	40	Methanol	28	66.00	56.24	36.50	33.7	1.65	2.01
30	75	85	55	Methanol	28	47.20	49.88	22.80	24.61	3.03	5.63
31	75	55	40	Methanol	28	51.20	45.67	21.00	22.05	2.97	3.90
32	75	85	25	Ethanol	40	39.70	44.29	21.20	32.38	3.43	1.02
33	100	25	40	Ethanol	28	19.30	23.52	3.20	1.96	2.98	5.59
34	75	55	40	Methanol	40	52.80	49.49	8.80	24.66	2.75	4.04
35	100	55	25	Methanol	28	18.30	25.81	3.30	5.44	5.66	6.38
36	100	25	40	Methanol	40	27.10	30.83	11.50	12.89	5.13	3.76
37	75	25	55	Methanol	28	30.30	44.09	15.30	24.55	1.95	2.08
38	75	85	25	Methanol	28	52.40	54.25	27.30	27.12	3.55	2.36
39	100	55	55	Ethanol	28	38.10	34.89	3.80	5.41	2.21	2.28
40	100	55	55	Methanol	28	32.80	32.7	11.60	7.21	5.07	5.88
41	75	55	40	Ethanol	40	49.80	46.81	32.90	30.17	1.98	2.69
42	100	85	40	Methanol	28	36.50	36.21	3.90	9.88	9.94	6.64
43	50	25	40	Ethanol	28	51.00	50.66	34.70	35.71	1.52	4.93
44	75	55	40	Ethanol	28	49.50	41.24	30.30	23.19	2.29	3.50
45	50	55	55	Ethanol	40	56.90	63.16	40.70	47.38	2.27	2.36
46	50	55	25	Methanol	28	49.90	62.89	33.60	33.59	1.28	1.53
47	50	55	55	Methanol	28	62.50	61.26	38.30	41.95	4.63	1.82
48	75	55	40	Ethanol	28	50.60	41.24	23.00	23.19	3.10	3.50
49	100	55	55	Ethanol	40	41.60	32.25	4.30	8.31	3.91	3.29
50	75	55	40	Methanol	40	41.90	49.49	17.40	24.66	3.40	4.04
51	75	55	40	Methanol	28	51.80	45.67	24.30	22.05	2.61	3.90
52	75	55	40	Methanol	40	53.30	49.49	23.30	24.66	2.95	4.04
53	75	55	40	Ethanol	28	46.60	41.24	25.30	23.19	2.48	3.50
54	75	25	25	Ethanol	28	21.80	29.05	5.70	14.05	17.76	8.72
55	75	85	55	Ethanol	40	50.30	39.08	37.00	20.15	2.59	4.65
56	100	85	40	Ethanol	28	23.10	32.94	1.50	10.17	3.05	2.92
57	75	25	55	Methanol	40	63.80	53.55	45.70	32.11	2.50	2.22
58	75	25	55	Ethanol	40	61.60	56.33	44.60	35.54	2.35	0.99
59	50	55	55	Ethanol	28	53.60	56.66	40.10	42.97	1.83	1.17
60	50	85	40	Ethanol	28	54.20	57.85	38.00	44.94	2.32	0.57

Note: X_1_: solvent concentration; X_2_: treated times; X_3_: treated temperatures; X_4_: types of solvents; X_5_: ultrasonic frequency; Y_1_: crude extraction; Y_2_: sediments after evaporation and left overnight; Y_3_: the yield of the precipitation.

## Data Availability

Data are contained within this article.

## Sample Availability

Samples of the compounds are not available from the authors.
